# Beyond Simple Grinding: Methylammonium Chloride Promotes Sustainable, Cylinder‐Free Mechanochemical Synthesis of Deferiprone

**DOI:** 10.1002/cssc.202500457

**Published:** 2025-07-14

**Authors:** Francesco Basoccu, Sara Piermarini, Tommaso Angelini, Massimiliano Mari, Edoardo Mariani, Alessandro Falchi, Andrea Porcheddu

**Affiliations:** ^1^ Dipartimento di Scienze Chimiche e Geologiche Università degli Studi di Cagliari Cittadella Universitaria Monserrato S.P. Monserrato‐Sestu Km 0,700 09042 Monserrato CA Italy; ^2^ Centro Ricerche‐Chiesi Farmaceutici S.p.A Largo Belolli 11/a 43126 Parma PR Italy

**Keywords:** APIs, deferiprone, green chemistry, mechanochemistry, thalassemia

## Abstract

Thalassemias are a group of inherited hemoglobinopathies that disrupt normal hemoglobin synthesis. Managing these conditions often involves regular blood transfusions and iron chelation therapy to mitigate iron overload. Among the available chelators, deferoxamine, deferasirox, and deferiprone, the latter stands out notably for its use when other drugs are ineffective or intolerable for patients. Although the synthesis of deferiprone is well‐documented, traditional methods typically involve lengthy reaction times and environmentally harmful conditions. This study investigates mechanochemistry as a more sustainable, efficient, and eco‐friendly alternative. Deferiprone is successfully synthesized and the production process is optimized by employing solid‐state techniques, thereby reducing reaction time, energy consumption, and environmental impact. These findings pave the way for cleaner and more sustainable manufacturing pathways for this critical agent in thalassemia management.

## Graphical abstract

Deferiprone is synthesized under solvent‐free mechanochemical conditions using ball milling. Methylammonium chloride and KOH enable efficient substitution of the oxygen atom in the maltol scaffold with a methylamine moiety, avoiding the limitations of methylamine solutions. The approach delivers good yields and shorter reaction times and eliminates toxic solvents, highlighting its sustainability.

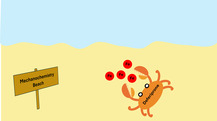

## 1 Introduction

Thalassemia is an inherited blood disorder characterized by abnormal hemoglobin production.^[^ ^1^, ^2^, ^3^, ^4^ ^]^ Most patients experience anemia of varying intensity because the disease diminishes both the production and lifespan of red blood cells.^[^ ^5^ ^]^ Classic anemic features, fatigue, pallor, and weakness, may be mild or pronounced and, at times, progress into more complex clinical pictures.^[^ ^6^, ^7^ ^]^

Thalassemia encompasses several forms, each with various subtypes arising from different alterations in hemoglobin synthesis.^[^ ^8^, ^9^, ^10^, ^11^ ^]^ Alpha‐thalassemia represents the most severe end of the spectrum, typically marked by profound anemia, splenomegaly, bone deformities, and iron deficiency overload.^[^ ^12^, ^13^, ^14^, ^15^, ^16^ ^]^ This complication is not inherent to thalassemia; it arises only following the repeated blood transfusions administered to manage the anemia. Each transfusion introduces additional iron, and unless it is removed, the metal gradually accumulates. Excess iron accumulates in critical organs, particularly the heart, liver, and endocrine glands, where it can progressively impair their function and become life‐threatening.^[^ ^17^, ^18^, ^19^ ^]^

**Figure 1:**
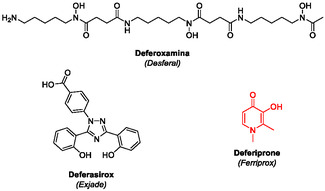
Commonly used iron chelators for managing thalassemia major.

Management, therefore, relies on a multifaceted strategy that combines regular transfusions with lifelong iron chelation, hematopoietic stem‐cell transplantation for selected patients, and comprehensive supportive measures to mitigate complications.^[^ ^12^, ^20^ ^]^ Deferoxamine (Desferal) and the oral agent deferasirox (Exjade, Jadenu) are the standard first‐line chelators.^[^ ^21^, ^22^, ^23^ ^]^ When these drugs are ineffective, poorly tolerated, or unavailable, clinicians switch to the oral chelator deferiprone (Ferriprox), which demands close hematologic and biochemical monitoring (**Figure** **1**).^[^ ^24^, ^25^, ^26^ ^]^ Deferiprone, known chemically as 3‐hydroxy‐1,2‐dimethylpyridine‐4(1H)‐one, was first approved in 1994 for the treatment of Thalassemia major and has been authorized for this use in the European Union for many years.^[^ ^27^, ^28^ ^]^ On October 14, 2011, it received approval from the USA through the FDA's accelerated regulatory approval process. The most common side effects include red‐brown urine, which indicates that iron is being excreted through urine, as well as nausea, abdominal pain, and vomiting. Less frequent but more severe side effects may include agranulocytosis and neutropenia.^[^ ^29^, ^30^ ^]^

**Scheme 1:**
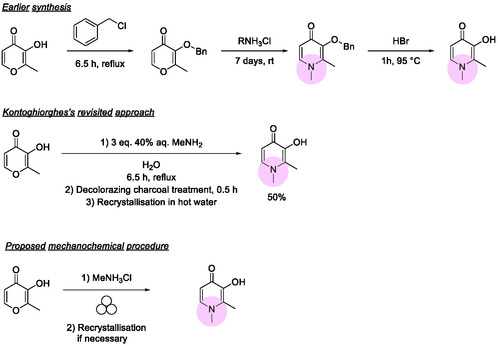
Reported methodologies for synthesizing deferiprone in solution, along with the mechanochemical approach proposed herein.

Deferiprone is synthesized via two main routes (**Scheme** **1**). Both methods initiate by masking the hydroxyl group as an ether. The protected intermediate is then coupled with the necessary primary amine, introduced as its ammonium salt, under basic conditions. Lastly, treatment with HBr removes the protecting group, regenerating the free alcohol.^[^ ^31^ ^]^ In 1987, Kontoghiorghes proposed a different methodology, preparing deferiprone through a single reaction step by reacting maltol with an excess of methylamine water.^[^ ^31^ ^]^

Although the published routes are conceptually simple, they share several shortcomings. The desired product is almost always accompanied by a persistent impurity, 1,2‐dimethyl‐4‐(methylimino)‐1,4‐dihydropyridin‐3‐ol. In addition, the reactions are typically run in solution at high temperatures, which can degrade the starting maltol and various intermediates, generating numerous aromatic and polymeric sugar byproducts.^[^ ^32^, ^33^, ^34^, ^35^ ^]^ Subsequent improvements have not eliminated the use of harsh reagents or extreme conditions for heating.^[^ ^36^, ^37^, ^38^, ^39^, ^40^ ^]^ For example, Orvig's method replaces HBr cleavage with catalytic hydrogenolysis, yet still requires prolonged reaction times and affords only a 53% yield of deferiprone, offering a slight practical advantage.^[^ ^39^ ^]^

To pursue a more sustainable and efficient approach, we adopted a mechanocatalytic approach. Building on Kontoghiorghes’ single‐step concept, we employed ball milling to combine the protected maltol with an excess of methylammonium salt, which releases methylamine in situ. This solid‐state method results in faster reactions and milder conditions and significantly reduces solvent usage.^[^ ^41^, ^42^, ^43^, ^44^ ^]^ It also replaces the flammable, corrosive methylamine solution (H225, H302, H314, H331, H335) with the much safer solid methylammonium chloride (H302).^[^ ^45^, ^46^ ^]^ Harnessing the intrinsic sustainability of mechanochemistry,^[^ ^47^, ^48^, ^49^, ^50^, ^51^, ^52^, ^53^, ^54^, ^55^, ^56^, ^57^ ^]^ we developed an efficient, solvent‐minimized pathway to deferiprone. The study further elucidates how gaseous methylamine is generated and behaves under solid‐state conditions, offering valuable mechanistic insight.^[^ ^49^, ^58^, ^59^ ^]^

## 2 Results and Discussion

### 2.1 Optimized Mechanochemical Route to Deferiprone (2)

The system's reactivity was evaluated by milling maltol (**1**) in the presence of methylammonium chloride to synthesize deferiprone (**2**). The synthesis of **2** generally involves the formation of the collateral product 1,2‐dimethyl‐4‐(methylimino)‐1,4‐dihydropyridin‐3‐ol (**3**) and sugar‐derived polymers (**4**). These initial experiments were conducted using a 10 mL ZrO_2_ jar containing two milling balls made from the same material (10 mm Ø, individual ball weight: 2.9845 g). Preliminary tests without a base did not convert the starting material into the desired product (**Table** **1**, entries 1 and 2).

**Table 1:** Optimization of **2** synthesis.

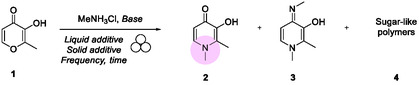							
Entry^a)^	Base [eq.]	Time	Liquid additive [*η*]	Solid additive [eq.]	Yield% 2	Yield% 3	Yield% 4
1b)	/	2	/	/	0	0	0
2b)	/	2	MeOH (0.3)	/	0	0	0
3	KOH (3.0)	2	/	/	28	11	21
4	KOH(3.0)	2	MeOH (0.3)	/	4	1	0
5c)	KOH (3.0)	2	/	/	25	8	16
6	KOH (6.0)	2	/	/	34	10	26
7	KOH (6.0)	3	/	/	42	17	25
8	KOH (6.0)	4	/	/	53	19	22
9d)	KOH (6.0)	4	B(OMe)_3_	/	54	19	22
10	KOH (6.0)	4	/	BHT (0.02)	52	17	26
11d)	KOH (6.0)	4	B(OMe)_3_	BHT (0.02)	59	20	16

a) Unless otherwise stated, all the reactions were run on a 2.0 mmol scale using **1** as the starting material, which was made by reacting with 3.0 eq. of MeNH_3_Cl in the presence or absence of a base. The experiments were performed using a 10 mL ZrO_2_ jar equipped with two milling balls (ZrO_2_, 10 mm Ø, weight of a single ball = 2.9845 g), which was shaken at 50 Hz using a Fritsch Mini Mill‐Pulverisette 23 milling device. The reaction time was expressed in terms of hours. All the bases employed were ground with a mortar and pestle before being used in a reaction unless they were already powdery in their composition. The presence of the liquid additive was expressed in terms of the *η* factor (ratio between the amount of liquid additive used in terms of μL and the total amount of reaction components in mg). The reaction yield refers to the molar yield calculated using HPLC analysis based on previously recorded calibration lines. The yield of **4** was calculated as the difference between the data collected for other reaction components.

b) 3.0 mmol of **1**were employed.

c) Starting material **1** was ground under ball milling conditions.

d) 0.35 eq. (B(OMe)_3_).

Both inorganic and organic bases were evaluated (for more details, please see section 4 in the Supporting Information). However, only KOH demonstrated significant performance, achieving a 28% deferiprone yield after 2 h under neat grinding conditions (Table 1, entry 3). Interestingly, the solvent used as the liquid additive had an adverse effect on the reaction outcome (Table 1, entry 4). Subsequently, these conditions were tested at various grinding frequencies, with 50 Hz identified as the optimal frequency for achieving a high conversion of **1** into **2**.^[^ ^60^ ^]^ Following the confirmation of optimized conditions using KOH as the base, a series of additional experiments were conducted to improve the yield of **2** further.

Considering that the reaction involves the in situ released gaseous methylamine and the solid particles of **1**, a smaller crystal size with a larger surface area is expected to enhance interactions with the gas (Table 1, entry 5). Nevertheless, no increase in yield was observed under these conditions. Doubling the loadings of MeNH_3_Cl and KOH raised the yield to 34% (Table 1, entry 6), and extending the milling time to 4 h drove the conversion of **1** to completion (Table 1, entries 7 and 8).^[^ ^61^ ^]^

As previously mentioned, the process typically involves the formation of sugar‐like polymers as byproducts.^[^ ^32^, ^33^ ^]^ Side products, chiefly **3** and **4**, arise when reaction intermediates follow competing pathways or react with atmospheric O_2_.^[^ ^62^ ^]^

Consequently, we investigated whether the combination of the complexing agent B(OMe)_3_ with the widely used antioxidant butylated hydroxytoluene (BHT) could inhibit both polymerization and the formation of by‐products **3**.^[^ ^40^ ^]^ Their action were assessed by adding them singularly and simultaneously (Table 1, entries 9–11). The optimal results were achieved using substoichiometric amounts of B(OMe)_3_ (0.35 eq.) and a catalytic quantity of BHT (0.02 eq.), resulting in a 59% yield of deferiprone after 4 h of milling (Table 1, entry 11).

### 2.2 Influence of the Counter‐Cation and Base Strength on the Maltol‐to‐Deferiprone Mechanosynthesis

During the mechanochemical conversion of **1** into **2**, two variables were found to be decisive: the identity of the accompanying cation and the intrinsic basicity of the additive.

To isolate the cation effect, we conducted solution trials in which maltol was stirred with an ethanolic solution of methylamine in the presence of common alkali‐metal chlorides (ESI, Section 5). Under these homogeneous conditions, neither NaCl nor KCl provided meaningful assistance; the yield of deferiprone plateaued at 10% and 9%, respectively, indicating that a benign counter‐ion alone cannot drive the reaction.

**Figure 2:**
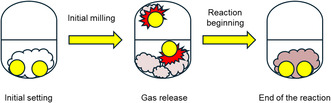
Steps in the mechanochemical synthesis of deferiprone. First, the initial milling step reduced the particle size of the reaction components, resulting in the release of gaseous MeNH_2_. The gas occupied the entire available volume (≈10 mL), marking the onset of the organic reaction.

The crucial role of base strength became clear when we moved back to the solid state. Because methylamine must be liberated from methylammonium chloride in situ, we hypothesized that stronger bases would accelerate gas release and, therefore, product formation. To test this, we charged a ZrO_2_ milling jar with methylammonium chloride, the base under investigation, and an inert filler (anhydrous Na_2_SO_4_) that preserved a constant filling volume.^[^ ^63^ ^]^ As milling proceeded, the base deprotonated MeNH_3_Cl, releasing methylamine into the jar's headspace; the gaseous amine was promptly absorbed by the surrounding maltol powder, initiating further reaction (**Figure** **2**).

A systematic screen of inorganic bases (Section 6, Supporting Information) confirmed the trend: only strong bases delivered full methylamine evolution. Hydroxides far outperformed carbonates and alkaline‐earth oxides, with potassium hydroxide emerging as the most efficient promoter when the mill was operated at 50 Hz. Reducing the frequency throttled methylamine production and, by extension, the formation of deferiprone.

In summary, efficient conversion of maltol to deferiprone hinges on a synergistic combination of an appropriate cation (potassium) and a genuinely strong base (KOH), which together ensure rapid and complete liberation of methylamine under mechanochemical conditions.

### 2.3 Reactivity of Maltol under Static Solid‐State Exposure to Methylamine: The Role of Added Cations

**Figure 3:**
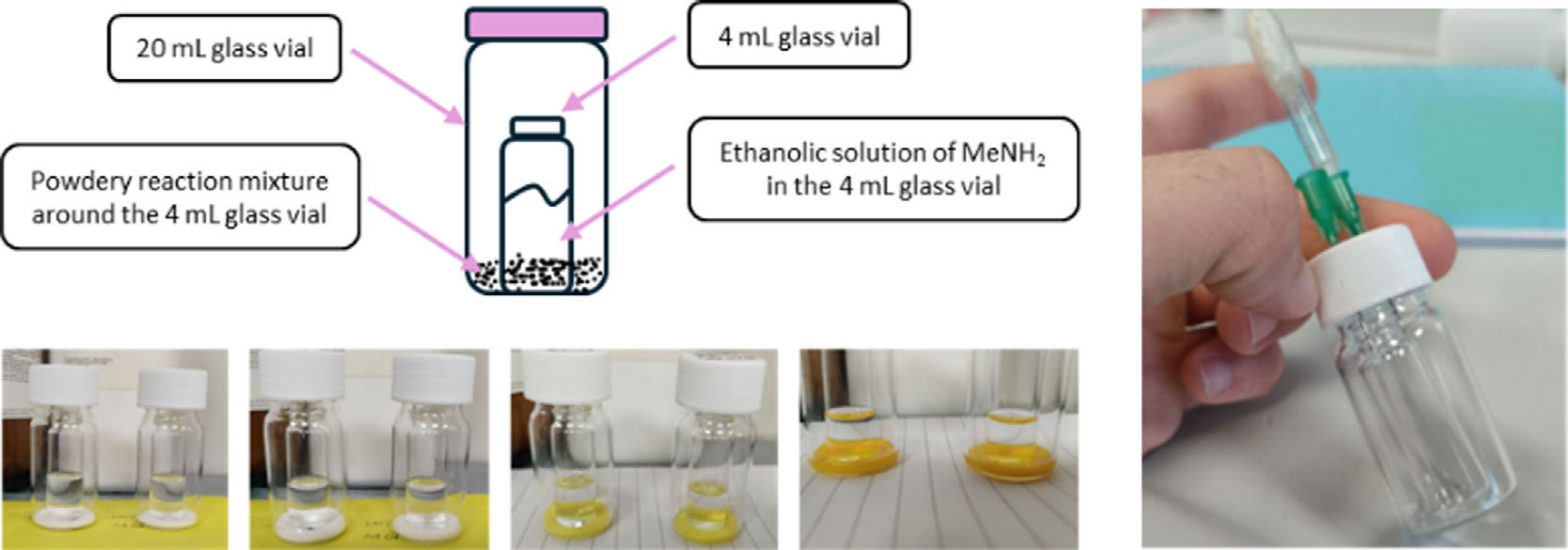
The reactions were performed in the presence of gaseous MeNH_2_ under static conditions. On the left is a general description of the setup used for running the reactions. The apparatus for the trial is depicted on the right under an argon atmosphere.

To investigate the specific role of inorganic cations, aside from the mechanical input of ball‐milling, we devised a “static‐gas” experiment (**Figure** **3**). Finely ground **1** was placed in a 20 mL glass vial, and a smaller open vial containing a 33% ethanolic solution of methylamine was positioned inside. As the amine gradually evaporated, its vapor diffused through the headspace and adsorbed onto the maltol powder. Identical trials were conducted with and without a weighed aliquot of an alkali‐metal chloride thoroughly premixed into the solid; all other variables were kept constant (Figure 3).

In the absence of extra cations, the reaction was extremely sluggish: after 24 h, we recovered only unreacted **1** (**Table** **2**, entries 1–6).

**Table 2:** Analysis of 1 reactivity in the absence or presence of a cation source under static solid‐state conditions.

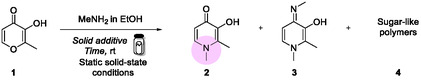						
Entrya)	Time	Solid additive [eq.]	Yield% 1	Yield% 2	Yield% 3	Yield% 4
1	2 h	/	100	/	/	/
2b)	2 h	/	100	/	/	/
3c)	2 h	/	100	/	/	/
4	1 d	/	100	/	/	/
5b)	1 d	/	100	/	/	/
6c)	1 d	/	100	/	/	/
7	2 d	/	61	12	9	18
8	2 d	NaCl (1)	17	26	13	44
9	2 d	KCl (1)	19	25	15	41
10	5 d	/	1	35	30	34
11	5 d	NaCl (1)	0	38	24	38
12	5 d	KCl (1)	0	36	31	33
13d)	5 d	/	3	38	36	23

a) Unless otherwise stated, all the reactions were run on a 2.0 mmol scale using **1** as the starting material, reacting with 3.0 eq. MeNH_2_ in EtOH in the presence or absence of a solid cation source. The experiments were run using a 20 mL glass vial equipped with a plastic lid containing a 4 mL glass vial charged with a solution of MeNH_2_ in EtOH. The reaction time was expressed in terms of hours. Before use, all the reaction components were ground with a mortar and pestle. The reaction yield refers to the molar yield calculated using HPLC analysis based on previously recorded calibration lines. The yield of **4** was calculated as the difference from the data collected for the other reaction components.

b) MeNH_2_ in H_2_O was used as the solvent.

c) MeNH_2_ in THF.

d) The reaction was carried out under an argon atmosphere.

When NaCl or KCl were introduced in the reaction environment, their influence on the static exposure became more evident (Table 2, entries 7–9): relative to the salt‐free control, Na^+^ or K^+^ increased maltol conversion by roughly 40% and almost doubled the yield of deferiprone. Hence, the cations accelerate productive ring opening early in the process rather than altering the ultimate equilibrium composition.

When left undisturbed for 5 days, instead, the starting material was almost entirely consumed, giving a complex mixture in which **2**, **3**, and **4** appeared in comparable amounts (Table 2, entry 10). Thus, methylamine vapor alone can open the **1** ring under static conditions; however, selectivity is poor, resulting in similar yields of products **2**, **3**, and **4**.

Introducing NaCl or KCl subtly but consistently shifted the outcome. Both salts raised the yield of **2**, and NaCl altered the **2**:**3** ratio in favor of the target drug (Table 2, entries 11–12). These changes imply that alkali‐metal cations interact with the reactive intermediates, most likely by stabilizing the anionic fragments that are formed during ring opening, and in doing so deter their diversion into polymeric side‐paths. Conducting the salt‐assisted experiment under an argon atmosphere led to a slight but consistent increase in the amounts of **2** and **3** (Table 2, entry 13). This suggests that traces of O_2_ otherwise facilitate the competing degradation pathway of reaction intermediates into sugar‐like polymeric by‐products.

Taken together, the data indicate that alkali‐metal cations funnel a larger fraction of the methylamine‐activated maltol into deferiprone by stabilizing intermediate anions and suppressing their degradation to sugar‐derived polymers. Under purely static conditions, the effect is kinetic: the cations do not change the final product blend when the system is allowed to run to completion, but they exert decisive control over the reaction rate and early‐stage selectivity.

### 2.4 Analyze How Na^+^ and K^+^ Influence the Solution‐Phase Conversion of Maltol

To determine whether alkali‐metal cations actively guide the methylamine‐driven conversion of **1** to **2** in solution, we performed a series of in‐solution experiments in which a 40 wt% aqueous MeNH_2_ solution was employed in an aqueous medium (**Table** **3**). At 100 °C, the reaction was essentially complete within 60 min, whereas halving the time left noticeable residual **1** (Table 3, entries 1–2). We further attenuated the conditions by reducing the process time to 15 min, thereby exposing subtler cation effects (Table 3, entry 3). Under this milder regime, three equivalents of KCl raised the yield of **2** by ≈10% and suppressed the formation of polymeric by‐product **4** (Table 3, entry 4). Notably, complexing K^+^ with 18‐crown‐6 offered no additional benefit (Table 3, entry 5). When the reaction was run with just one equivalent of MeNH_2_ for 30 min, one equivalent of KCl again delivered the highest conversion (Table 3, entries 6–7). In contrast, an identical amount of NaCl conferred a slight advantage, and sub‐stoichiometric doses of either salt were ineffective, indicating that a threshold concentration of K^+^ is required (Table 3, entries 8–10). Changing the solvent to methanol, in which KCl is practically insoluble, largely erased the potassium benefit, and replacing KCl with the readily soluble NaI paradoxically cut the yield of **2** to only 7% (Table 3, entries 11–14). Taken together, these observations show that potassium, rather than mere ionic strength or salt solubility, plays a specific, rate‐ and selectivity‐enhancing role in steering maltol toward deferiprone in solution.

**Table 3:** Analysis of 1 reactivity in the absence or presence of a cation source under solution conditions.

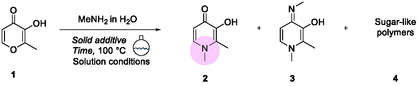							
Entry^a)^	Time [min]	MeNH_2_ [eq.]	Solid additive [eq.]	Yield% 1	Yield% 2	Yield% 3	Yield% 4
1	60	3.00	/	1	75	16	8
2	30	3.00	/	13	58	12	17
3	15	3.00	/	34	47	8	11
4	15	3.00	KCl (3.00)	31	55	9	5
5b)	15	3.00	KCl (3.00)	25	57	10	8
6	30	1.00	/	66	24	0	10
7	30	1.00	KCl (1.00)	53	35	2	10
8	30	1.00	NaCl (1.00)	72	19	0	9
9	30	1.00	KCl (0.05)	63	18	0	19
10	30	1.00	NaCl (0.05)	71	14	0	15
11c)	30	3.00	/	38	19	10	33
12c)	30	3.00	NaCl (3.00)	58	20	6	26
13c)	30	3.00	KCl (3.00)	53	25	11	11
14c)	30	3.00	NaI (3.00)	63	7	0	30

a) Unless otherwise stated, all the reactions were run on a 2.0 mmol scale using **1** as the starting material, which was made by reacting with MeNH_2_ in H_2_O and distilled water (4 mL) in the presence or absence of a solid cation source. The experiments were run using a 20 mL crimped glass vial equipped with an aluminium lid. The reaction yield refers to the molar yield calculated using HPLC analysis based on previously recorded calibration lines. The yield of **4** was calculated as the difference from the data collected for the other reaction components.

b) 18‐crown‐6 (0.5 eq.) was added to the reaction mixture.

c) MeNH_2_ in MeOH (40%wt) and MeOH (4 mL) were used.

Taken together, our results position the mechanochemical protocol as the most attractive entry to **2**. With only 0.35 equiv of B(OMe)_3_ and a catalytic 0.02 equiv of BHT, the solid‐state reaction delivers 59% isolated yield in just 4 h, without resorting to bulk solvent or prolonged heating.

We then benchmarked this route against our best‐performing solution experiment. Although the solution run reaches a somewhat higher yield, it does so at the cost of a markedly larger mass of auxiliary materials and solvent and therefore generates substantially more waste. A quantitative comparison using the Environmental Factor (EF) underscores the point: the EF for the solid‐state protocol is decisively lower, confirming that the mechanochemical approach is not only synthetically efficient but also the more sustainable choice.

The EF values derived from the two methodologies clearly underscore the benefits of mechanochemistry: 8.03 for the mechanochemical process, in contrast to 21.25 for the in‐solution approach and 132.91 for the protocol reported by Orvig.^[^ ^39^ ^]^ The findings highlight a significant reduction in waste achieved through mechanochemistry, in accordance with the principles of green chemistry. Section 8 of the Supporting Information provides a detailed breakdown of the calculations and additional supporting data.

While quantitative metrics are crucial for assessing sustainability, the advantages of green chemistry extend far beyond what numbers alone can convey. The primary benefit of this approach is the replacement of gaseous or aqueous methylamine with a safer solid alternative. This substitution significantly enhances process safety, reduces handling risks, and eliminates the need for specialized infrastructure for storing and transporting hazardous reagents. Consequently, it minimizes operator exposure while improving the overall practicality of the synthesis.

The method offers substantial economic and environmental advantages by eliminating the need for autoclaves and other pressurized equipment. This reduction leads to lower operational costs, simplifies scale‐up processes, and expands access to deferiprone synthesis, as high‐pressure apparatus is no longer necessary. Additionally, the process has a smaller footprint, allowing the reaction to be conducted in compact workspaces rather than large‐scale facilities.

Ultimately, by minimizing energy‐intensive cooling and excessive water usage, this approach conserves resources and reduces CO_2_ emissions, further aligning the synthesis with the fundamental principles of green and sustainable chemistry. This holistic perspective emphasizes that sustainability encompasses not only efficiency and waste reduction, but also safety, accessibility, and energy‐conscious design, fundamental pillars of a truly green chemical industry process.

### 2.5 Reaction Mechanism

The data suggest that under solid‐state conditions, the reaction outcome is primarily influenced by three factors: the choice of base, the specific cation present, and the use of a liquid additive. These findings collectively identify KOH as the optimal base for this application. Potassium hydroxide quickly releases gaseous MeNH_2_ from solid MeNH_3_Cl while simultaneously promoting deferiprone formation. However, in solution, its positive effect is reduced, likely because solvent coordination competes with K^+^‐substrate interaction.

**Figure 4:**
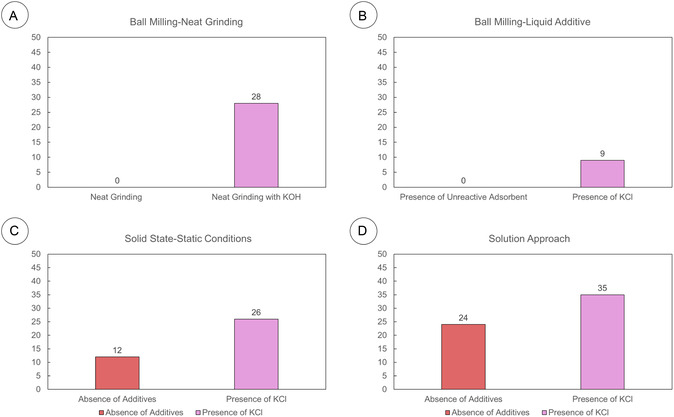
Comparison between solid and solution techniques. A) Comparison of the grinding approach using other bases and KOH. B) Comparison of grinding approaches using a liquid additive in the absence and presence of a solid adsorbent. C) Comparison of the static solid‐state approach in the absence and presence of potassium chloride. D) Comparison of the in‐solution approach in the absence and presence of potassium chloride.

**Scheme 2:**
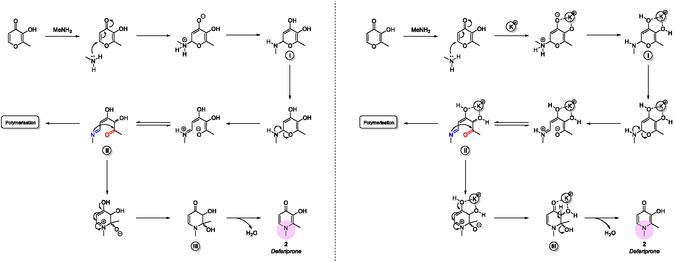
Proposed reaction mechanism and scavenger activity of the K^+^ cation.

Collectively, these findings help us understand the dual role of K^+^ in both reactivity (**Figure** **4**) and mechanism (**Scheme** **2**). The reaction begins with methylamine's nucleophilic attack on the maltol ring, producing intermediate **I**. This is followed by resonance‐assisted ring opening, which generates intermediate **II**, characterized by a nucleophilic imine and an electrophilic C‐6 carbonyl. This ambident nature makes **II** prone to oligomerization, resulting in a polymeric by‐product. At this point, K^+^ influences the rate of the competing polymerization pathway. Previous studies indicate that complexation of the 3,4‐dihydroxy motif of maltol can direct the reaction towards the desired product **2** by mitigating the electron‐withdrawing effect of the carbonyl on the amino group.^[^ ^40^ ^]^ While covalent boronate esters achieve this through strong B—O bonds, a similar, albeit weaker, hard–hard interaction between K^+^ and the C‐4 carbonyl can still lead to measurable rate acceleration and selectivity, even if the effect is less pronounced than that of a true covalent linkage.

**Scheme 3:**
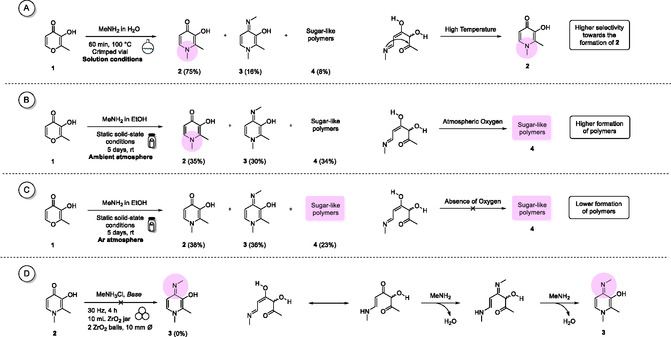
The proposed mechanism for the formation of impurities under various conditions is screened. A) Analysis of methylamine reactivity under solution conditions. B) Analysis of maltol reactivity under solid‐state conditions and ambient atmosphere. C) Analysis of the reactivity of maltol under solid‐state conditions in an argon atmosphere. D) Analysis of deferiprone reactivity concerning the formation of impurity **3**.

**Scheme** **3** illustrates the collateral pathways identified under the various reaction regimes examined. The reaction became significantly more selective when the synthesis was conducted in a solution at elevated temperatures in a sealed, pressurized vial with an aqueous stream of methylamine. However, this advantage comes at the cost of harsher conditions and a more hazardous reagent. In contrast, allowing gaseous MeNH_2_ to diffuse gently over solid maltol in an open system resulted in a broader product distribution, suggesting that milder static solid‐state conditions favor the formation of unwanted side‐products. Oxygen also proved to be a subtle player: repeating the static solid‐state experiment under an inert atmosphere reduced the amount of polymeric material by roughly ten per cent, indicating that O_2_ modestly accelerates the competing polymerization pathway. Finally, the origin of byproduct **3** was traced mainly to excess methylamine attacking transient intermediates rather than to nucleophilic attack on the highly robust deferiprone ring. These observations underscore a familiar yet instructive trade‐off: the tighter control provided by harsher, less sustainable conditions must be weighed against the greener but less selective nature of the milder protocols.

## 3 Conclusions

Building on existing literature, the synthesis of deferiprone through a mechanochemical approach was systematically investigated. This method enabled the process to occur under milder reaction conditions, providing a safer alternative to conventional methylamine solutions. Notably, the presence of cations enhances maltol's reactivity, significantly influencing the reaction pathway in solid‐state conditions. Interestingly, although this effect seemed to diminish, likely due to complexation in aqueous solvents, it was also observed in solution‐phase reactions. Furthermore, a thorough analysis of gas release and maltol reactivity enabled the identification of optimal reaction conditions for the entire process. This study clarified the impact of solution temperature and the role of mechanical forces in mechanochemical processes.

## Conflict of Interest

The authors declare no conflict of interest.

## Author Contributions

**Francesco Basoccu**: wrote the first draft of the article. **Francesco Basoccu** and **Andrea Porcheddu**: conceptualized the idea and revised the manuscript's drafts. **Francesco Basoccu**: performed the syntheses and collected the data. **Francesco Basoccu**, **Sara Piermarini**, **Tommaso Angelini**, **Massimiliano Mari**, **Edoardo Mariani**, and **Alessandro Falchi**: analyzed the data. **Andrea Porcheddu**: supervised the work. All authors contributed to the draft corrections and finalization.

## Supporting information

Supplementary Material

## Data Availability

The data that support the findings of this study are available in the supplementary material of this article.
