# Supplementary material for: Beyond Simple Grinding: Methylammonium Chloride Promotes Sustainable, Cylinder‐Free Mechanochemical Synthesis of Deferiprone
Source: ChemSusChem. 2025 Jul 14;18(16):e202500457. doi: 10.1002/cssc.202500457 (PMC12330328; doi:10.1002/cssc.202500457)
Supplement: Supplementary file 1 — Supplementary Material [file CSSC-18-e202500457-s001.pdf]

Supporting Information

Index

1. Materials and Methods 2
2. Role of the frequency chosen on the reaction outcome 3
3. Role of the time chosen on the reaction outcome 4
4. Screening of bases for the optimisation of Deferiprone synthesis under ball milling conditions 6
5. Influence of cations in a Liquid Assisted Grinding approach 7
6. Optimisation of the gas release under ball milling conditions 8
7. Calculation of gas release under milling conditions 9
8. Green Metrics 10

1. Materials and Methods

Commercially available reagents were purchased from Sigma-Aldrich, Alfa-Aesar, TCI Europe and used as received. Chemical reactions were carried out using Fritsch Pulverisette 23 Mini mill apparatus. The reagents were milled using a ZrO_2 grinding jar (10 mL) equipped with 2 balls (10 mm Ø, weight of a single ball= 2.9845 g) of the same material. For the analysis under static solid conditions, the reagents were previously ground with a mortar and a pestle and then mixed in a glass vial (20 mL) in which a smaller glass vial (4 mL) containing a 33% $MeNH_2$ in EtOH was placed. For the analysis of the gas release, it was checked the weight of the jar with the reagents loaded before and after the milling process for evaluating the amount of gas release as a difference between these two values. These parameters were applied if not stated otherwise. Retention times of different compounds were determined by injecting pure compound under identical conditions. Yields refer to the values detected on the HPLC analysis by using the proper calibration lines. The instrument used for the general HPLC method was a Waters Acquity Waters Micromass ZMD quadrupole mass spectrometer equipped with the column Acquity UPLC CSH C18 1.7um 2.1x50mm. The compounds have been detected with DAD Agilent, Mass Spectrometer detector at a wavelength of 210-400 nm with a constant temperature of 40 °C. The injection volume has been maintained at 10-2 µl with a constant flux of 1 ml/min. The eluent employed were an aqueous buffer pH 2.0 (EDTA dihydrate 0.03%, Sodium Octanesulfate 0.04%, NaH_2PO_4 monohydrate 1,2%) combined with Methanol HPLC grade. The ratio between the two eluents were 95%/5% respectively. The gradient was isocratic. The retention times can be described as follows:

Maltol: R_t= 2.82 min

Deferiprone: R_t= 5.987 min

Impurity C: R_t= 11.622 min

Sample Concentration: 75.0-100.0 mg of crude solid mixture dissolved into 250 mL of aqueous buffer.

2. Role of the frequency chosen on the reaction outcome

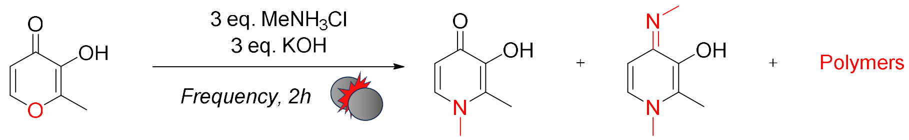

1 2 3 4

Table S1. Screening of the reaction frequency.

Entry	Frequency (Hz)	Yield% **1**	Yield% **2**	Yield% **3**	Yield% **4**
1	25	91%	2%	1%	6%
2	30	83%	13%	4%	0%
3	35	72%	6%	8%	14%
4	40	64%	18%	7%	11%
5	45	55%	26%	10%	9%
**6**	**50**	**40%**	**28%**	**11%**	**21%**

Otherwise stated, all the reactions were run on a 2.0 mmol scale using **1** as starting material which was made reacting with 3.0 eq. of $MeNH_3Cl$ in the presence of 3.0 eq. of KOH for 2 hours. The processes were run by using a 10 mL ZrO_2 jar equipped with 2 ZrO_2 milling balls (10 mm Ø) which was agitated at 50 Hz through a Mini Mill-Pulverisette 23 milling device developed by Fritsch. KOH was ground with a mortar and a pestle before being used in a reaction. The yield of the reactions for were calculated through HPLC on the basis of the calibration lines previously recorded. The yield of the sugar-like polymers (**4**) was calculated as a difference from the data collected from the other reaction components.

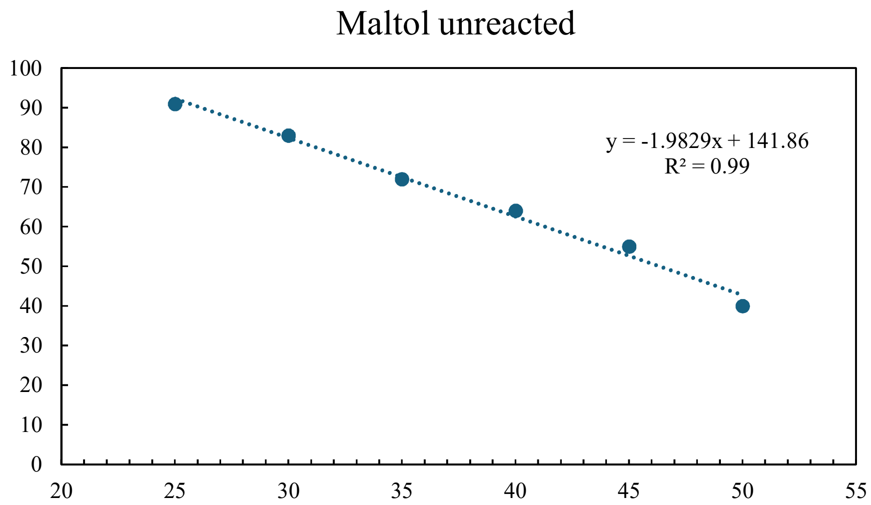

Graph S1. Percentage of maltol unreacted as a function of the frequency used for the milling process.

3. Role of the time chosen on the reaction outcome

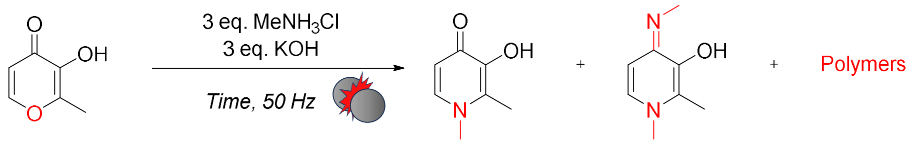

1 2 3 4

Table S2. Screening of the reaction frequency.

Entry	Time (min)	Yield% **1**	Yield% **2**	Yield% **3**	Yield% **4**
1	20	92%	6%	2%	0%
2	40	81%	12%	5%	2%
3	60	74%	13%	5%	9%
4	80	67%	21%	8%	4%
5	120	40%	28%	11%	21%
6	180	42%	31%	9%	18%

Otherwise stated, all the reactions were run on a 2.0 mmol scale using **1** as starting material which was made reacting with 3.0 eq. of $MeNH_3Cl$ in the presence of 3.0 eq. of KOH for 2 hours. The processes were run by using a 10 mL ZrO_2 jar equipped with 2 ZrO_2 milling balls (10 mm Ø) which was agitated at 50 Hz through a Mini Mill-Pulverisette 23 milling device developed by Fritsch. KOH was ground with a mortar and a pestle before being used in a reaction. The yield of the reactions for were calculated through HPLC on the basis of the calibration lines previously recorded. The yield of the sugar-like polymers (**4**) was calculated as a difference from the data collected from the other reaction components.

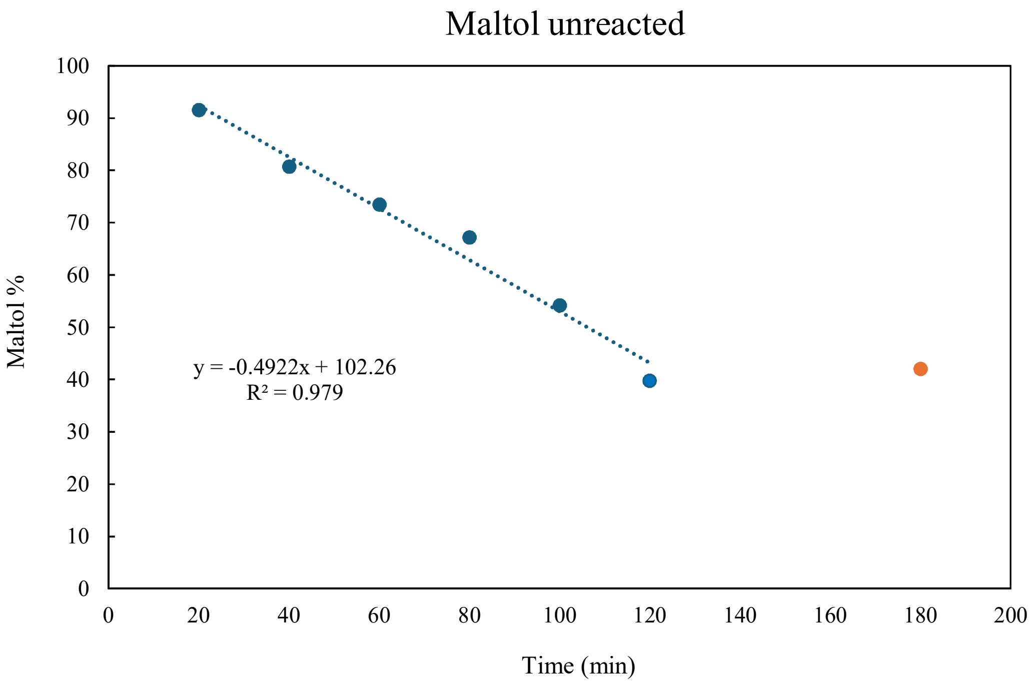

Graph S2. Analysis of consumption rate of maltol as a function of time.

The data collected at 180 minutes was labelled in orange due to the absence of linearity in terms of maltol consumption. This event is probably due to the absence of reactivity of maltol under this partial pressure of methylamine which brought us to investigate the reaction with a higher excess of methylamine precursor as reported in the manuscript.

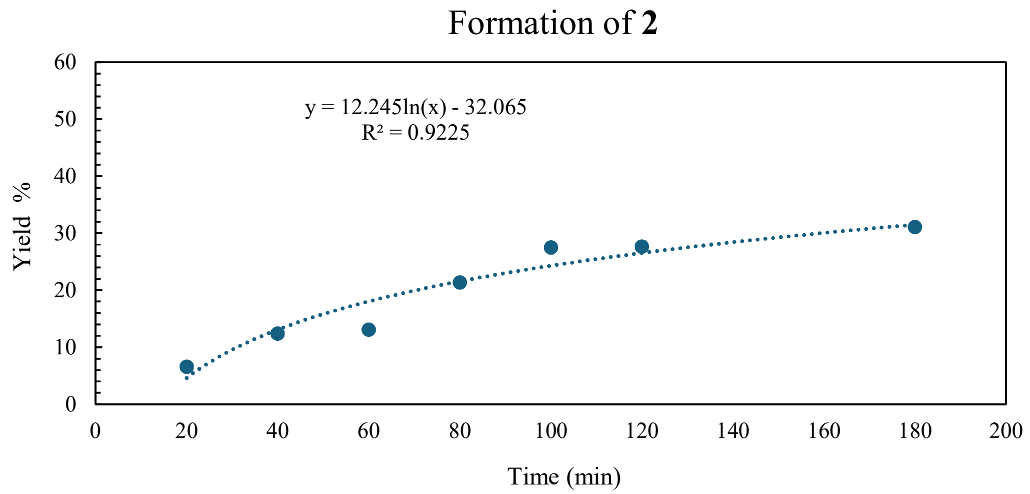

Graph S3. Yield of deferiprone as a function of time.

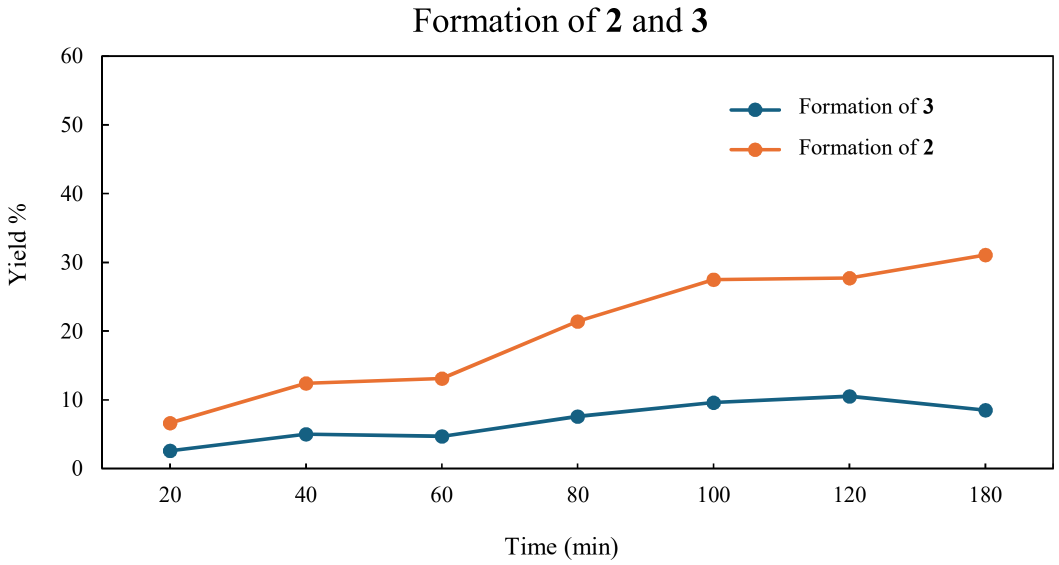

Graph S4. Comparison between the yields of **2** and **3**.

The ratio between the desired product **2** and the subproduct **3** remained constant over the time screening with an average value of 2.69.

4. Screening of bases for the optimisation of Deferiprone synthesis under ball milling conditions

Table S3. Optimisation of Deferiprone (**2**) synthesis.

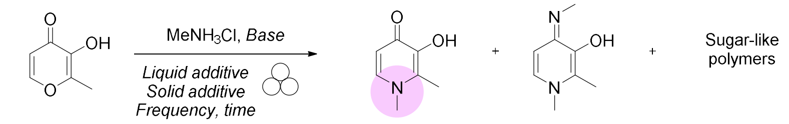

1 **2** **3** **4**

Entry	Base (eq.)	Time	Liquid additive (η)	Solid additive (eq.)	Yield% **2**	Yield% **3**	Yield% **4**
1[a]	/	2	/	/	/	/	/
2[a]	/	2	MeOH (0.3)	/	/	/	/
3	Na_2CO_3 (3.0)	2	/	/	1	/	/
4	K_2CO_3 (1.5)	2	/	/	/	/	/
5	$K_3PO_4 \cdot H_2O$ (3.0)	2	/	/	/	/	/
6	Cs_2CO_3 (1.5)	2	/	/	5	1	18
7	$LiOH \cdot H_2O$ (3.0)	2	/	/	/	/	/
8	KOH (3.0)	2	/	/	28	11	21
9	KOH(3.0)	2	MeOH (0.3)	/	4	1	/
10	$Mg(OH)_2$ (1.5)	2	/	/	/	/	/
11	$Ba(OH)_2$ (1.5)	2	/	/	1	/	/
12	DIPEA (3.0)	2	/	/	/	/	/
13	Imidazole (3.0)	2	/	/	/	/	/
14[b]	KOH (3.0)	2	/	/	25	8	16
15	KOH (6.0)	2	/	/	34	10	26
16	KOH (6.0)	3	/	/	42	17	25
17	KOH (6.0)	4	/	/	53	19	22
18[c]	KOH (6.0)	4	$B(OMe)_3$	/	54	19	22
19	KOH (6.0)	4	/	BHT (0.02)	52	17	26
**20[c]**	**KOH (6.0)**	**4**	**$B(OMe)_3$**	**BHT (0.02)**	**59**	**20**	**16**
21	Borax (1.5)	2	/	/	8	/	/

Unless otherwise stated, all the reactions were run on a 2.0 mmol scale using **1** as the starting material, which was made reacting with 3.0 eq. of $MeNH_3Cl$ in either the presence or absence of a base. The experiments were run by using a 10 mL ZrO_2 jar equipped with 2 ZrO_2 milling balls (10 mm Ø, weight of a single ball= 2.9845 g), which was agitated at 50 Hz through a Mini Mill-Pulverisette 23™ milling device developed by Fritsch. The reaction time is expressed in terms of hours. All the bases employed were ground with a mortar and a pestle before being used in a reaction unless already powdery in their composition. The presence of liquid additive was expressed in terms of η factor (ratio between the amount of liquid additive used in terms of µL and the total amount of reaction components in mg). The yield of the reactions refers to the molar yield calculated using HPLC analysis based on calibration lines previously recorded. The yield of the sugar-like polymers (**4**) was calculated as a difference from the data collected for the other reaction components. [a] 3.0 mmol of **1** were employed. [b] Starting material **1** was previously ground under ball-milling conditions. [c] 0.35 eq. of $B(OMe)_3$ were employed.

5. Influence of cations in a Liquid Assisted Grinding approach

Table S4. Analysis of maltol reactivity in either the absence or presence of a source of cations by using $MeNH_2$ in EtOH under mechanochemical conditions.

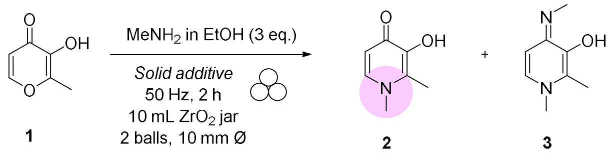

Entry	Time (h)	Solid additive (eq.)	Yield% **2**	Yield% **3**
1[a]	2	Silica	/	/
2	2	LiCl (3)	/	/
**3**	**2**	**NaCl (3)**	**10**	**3**
**4**	**2**	**KCl (3)**	**9**	**5**
5	2	$MgCl_2$ (3)	/	/
6	2	$CaCl_2$ (3)	/	/

Unless otherwise stated, all the reactions were run on a 2.0 mmol scale using **1** as the starting material, which was made reacting with 3.0 eq. of $MeNH_2$ in EtOH in the presence of a solid source of cations. The experiments were run by using a 10 mL ZrO_2 jar equipped with 2 ZrO_2 milling balls (10 mm Ø, weight of a single ball= 2.9845 g), which was agitated at 50 Hz through a Mini Mill-Pulverisette 23™ milling device developed by Fritsch. The yield of the reactions refers to the molar yield calculated using HPLC analysis based on calibration lines previously recorded. [a] 600.00 mg of silica was employed for evaluating the reactivity between maltol **1** and $MeNH_2$ in EtOH without any cation source.

6. Optimisation of the gas release under ball milling conditions

Table S5. Evaluation of the gas released from grinding the chosen base and $MeNH_3Cl$.

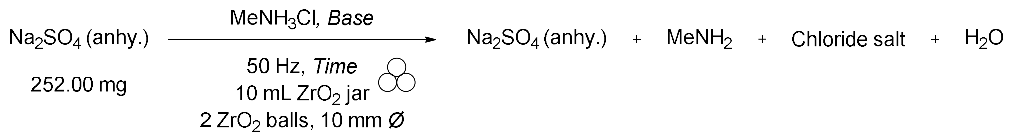

Entry	Time (min)	Base (eq.)	Amount of gaseous $MeNH_2$ released (mmol)
1	20	Na_2CO_3 (1)	1.09
2	60	MgO (1)	0.13
3	20	CaO (1)	1.80
4	60	CaO (1)	2.74
5	60	SrO (1)	5.06
**6**	**20**	**KOH (1)**	**6.00**
7[a]	20	KOH (1)	5.15
8	20	$Ca(OH)_2$ (0.5)	0.46
9	20	$Sr(OH)_2$ (0.5)	3.05
10	20	$Ba(OH)_2$ (0.5)	5.91
11	20	$Na_2B_4O_7{\cdot}10\,H_2O$ (1)	0.02

Unless otherwise stated, all the reactions were run by milling 252.00 mg of anhydrous Na_2SO_4, 6.00 mmol of $MeNH_3Cl$, and the chosen base by using a 10 mL ZrO_2 jar equipped with 2 ZrO_2 milling balls (10 mm Ø, weight of a single ball= 2.9845 g) which was agitated at 50 Hz through a Mini Mill-Pulverisette 23™ milling device developed by Fritsch. The amount of gas released was evaluated by accurately weighing all reaction components before and after the reaction. In the latter case, the final crude mixture was thoroughly dried on a static dryer before checking the final weight of the solid reaction mixture. [a] The reaction was run at 25 Hz.

7. Calculation of gas release under milling conditions

Herein it is depicted the general scheme employed for calculating the amount of gas released during the grinding of methylammonium chloride in the presence of a base. For simplifying the discussion, it has been analysed the specific case of KOH.

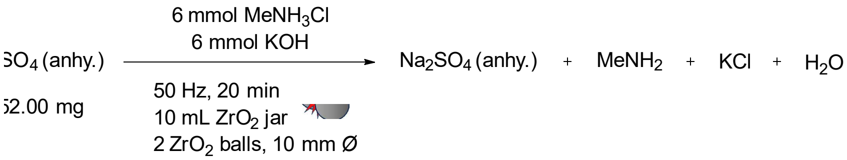

Reaction conditions	Reagents	Stoichiometry	mmol	Molecular weight	Theoretical amount	Real quantities involved	Real amount of mmol	Amount of unreacted material
10 mL ZrO_2 Jar<br>2 balls, 10 mm Ø<br>20 min, 50 Hz<br>Neat grinding	Na_2SO_4 Anhydrous	/	/	/	252.00 mg	263.4 mg	/	263.4 mg
	$MeNH_3Cl$	3	6 mmol	67.52	405.12 mg	410.8 mg	6.08 mmol	2.0 mg
	KOH (ground)	3	6 mmol	56.11	336.66 mg	339.3 mg	6.05 mmol	/
	$MeNH_2$ (gaseous)	3	6 mmol	31.10	186.6 mg	188.2 mg	6.05 mmol	/
	KCl	3	6 mmol	74.55	447.3 mg	450.8 mg	6.05 mmol	/

Jar tare with balls	Difference in weight before and after grinding	Real amount of solid material still present inside the jar	Total amount of water generated from the grinding
78.2723 g	229.0 mg	783.2 mg	108.0 mg
Jar tare with balls and reagents before grinding	Exceeding weight from gas release	Water evaporated during the grinding	mmol of water released
79.2845 g	40.8 mg	39.0 mg	6.00
Jar tare after grinding	Theoretical amount of solid material still present inside the jar	Amount of water still present in the in the solid material after grinding	**mmol of $MeNH_2$ released**
79.0555 g	822.2 mg	69.0 mg	**6.00**

The set-up consisted in accurately weighing the empty jar, all the reaction components, the jar loaded with reaction components before the grinding step and the jar loaded with reaction components after the grinding step. With these data, it was possible to calculate the amount of gas released on the basis of the stoichiometry of the reaction. In this specific case, there was a higher amount of milligrams missing after the reaction. This was attributed to the possible evaporation of some newly generated water during the impacts used for the reaction. This quantity detected matches with the difference between the theoretical amount of solid material still present inside the jar and the real amount of solid material detected after the grinding (the values have been highlighted in green in the table). From these calculations, it is possible to estimate also the quantity of water that was possible to weigh after the reaction leading to the calculation of total amount of water released from the process. This is equal to 6.00 mmol which demonstrates how the release of $MeNH_2$ was quantitative from this process. For all the other cases reported in the manuscript, it was applied a comparable estimation.

8. Green Metrics

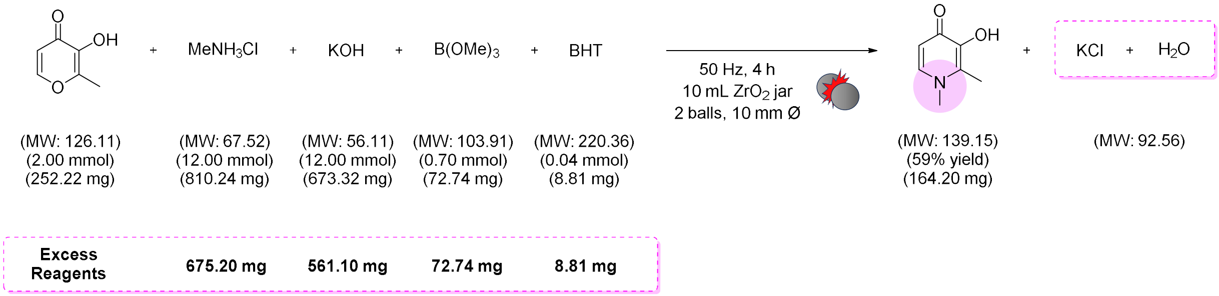

Scheme S1. Mechanochemical preparation of **2**.

Calculation of the Environmental Factor

$$\textbf{E}\text{nvironmental }\textbf{F}\text{actor} = \frac{\text{Mass of total waste}}{\text{Mass of desired product}} = \frac{675.20 + 561.10 + 72.74 + 8.81}{164.20} = \mathbf{8.03}$$

The Eco-scale Score for the mechanochemical preparation of Deferiprone (2)

EcoScale: 100 – sum of penalty points

Table S6. Calculation of Ecoscale score[a]

**Reagents**	**MF**	**MW**	**mg**	**mmol**	**Equiv.**
Maltol	$C_6H_6O_3$	126.11	252.2	2.00	1.00
Methylammonium Chloride	CH_5N . HCl	67.52	810.2	12.00	6.00
Potassium Hydroxide	KOH	56.11	673.2	12.00	6.00
Trimethyl Borate	$C_3H_9BO_3$	103.91	72.7	0.70	0.35
BHT	$C_{15}H_{24}O$	220.35	8.8	0.04	0.02
**Product**	**MF**	**MW**	**mg**	**mmol**	**Yield**
Deferiprone	$C_7H_9NO_2$	139.15	164.2	1.18	59%

**Entry**	**Parameters**	**Penalty Points**
1	Yield (59%)	-20
2	Price/availability	-3
3	Safety	-20
4	Technical set-up (Common set-up)	0
5	Temperature/time (r.t.; < 24 h)	-1
6	Work-up and purification (Crystallisation)	-1
	**EcoScale Score**	55

[a]Values calculated using the eco scale calculator software available at the link: http://ecoscale.cheminfo.org/calculator

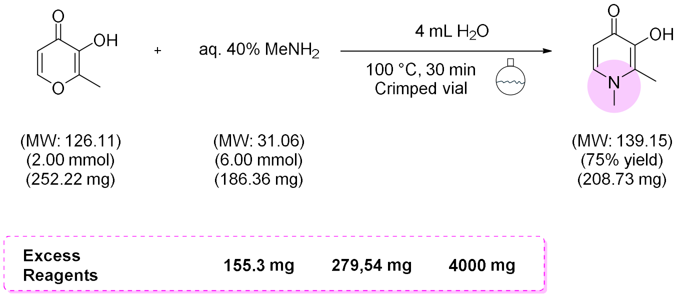

Scheme S2. In-solution preparation of **2**.

Calculation of the Environmental Factor

$$\textbf{E}\text{nvironmental }\textbf{F}\text{actor} = \frac{\text{Mass of total waste}}{\text{Mass of desired product}} = \frac{155.30 + 279.54 + 4000}{208.73} = \mathbf{21.25}$$

The Eco-scale Score for the in-solution preparation of Deferiprone (2)

EcoScale: 100 – sum of penalty points

Table S7. Calculation of Ecoscale score[a]

Reagents	MF	MW	mg	mmol	Equiv.
Maltol	$C_6H_6O_3$	126.11	252.2	2.00	1.00
Aq. Methylammine	CH_5N	31.06	186.3	6.00	3
Water	H_2O	18.02	4373.0	/	/
**Product**	**MF**	**MW**	**mg**	**mmol**	**Yield**
Deferiprone	$C_7H_9NO_2$	139.15	208.7	1.50	75%

Entry	Parameters	Penalty Points
1	Yield (75%)	-13
2	Price/availability	-11
3	Safety	-10
4	Technical set-up (Pressure equipment >1 atm)	-3
5	Temperature/time (heating; < 1 h)	-2
6	Work-up and purification (Cooling to RT, Crystallisation)	-1
	**EcoScale Score**	60

[a]Values calculated using the eco scale calculator software available at the link: http://ecoscale.cheminfo.org/calculator

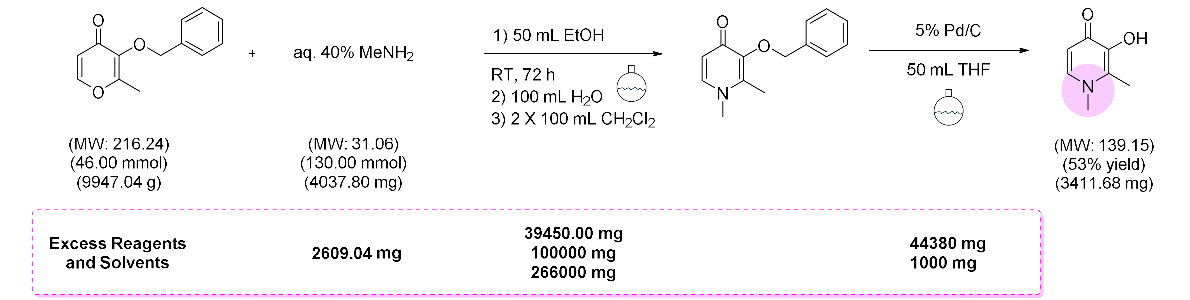

Scheme S3. In-solution preparation of **2** as reported by Orvig (W. O. Nelson, T. B. Karpishin, S. J. Rettig, C. Orvig, *Can. J. Chem.* **1988**, 66, 123-131).

Calculation of the Environmental Factor

$$\textbf{E}\text{nvironmental } \textbf{F}\text{actor} = \frac{\text{Mass of total waste}}{\text{Mass of desired product}} = \frac{2609.04 + 39450.00 + 100000.00 + 266000.00 + 44380.00 + 1000.00}{3411.68} = \mathbf{132.91}$$

The Eco-scale Score for the in-solution preparation of Deferiprone (2)

EcoScale: 100 – sum of penalty points

Table S8. Calculation of Ecoscale score[a]

Reagents	MF	MW	mg	mmol	Equiv.
3-(benzyloxy)-2-methyl-4H-pyran-4-one	$C_{13}H_{12}O_3$	216.24	9947.04	46.00	1.00
Aq. Methylammine	CH_5N	31.06	186.3	130.00	3
Ethanol	C_2H_6O	46.07	39450.00	/	/
Water	H_2O	18.02	100000.00	/	/
Dichloromethane	CH_2Cl_2	84.93	266000.00	/	/
Palladium on carbon	Pd/C	/	1000.00	/	/
Tetrahydrofuran	C_4H_8O	72.11	44380	/	/
**Product**	**MF**	**MW**	**mg**	**mmol**	**Yield**
Deferiprone	$C_7H_9NO_2$	139.15	3411.68	24.5	53%

Entry	Parameters	Penalty Points
1	Yield (53%)	-23.5
2	Price/availability	-14
3	Safety	-40
4	Technical set-up (Pressure equipment >1 atm)	-3
5	Temperature/time (room temperature; >24 h)	-1
6	Work-up and purification (Crystallisation)	-1
	**EcoScale Score**	17.5

[a]Values calculated using the eco scale calculator software available at the link: http://ecoscale.cheminfo.org/calculator
